# Knowledge and vaccination acceptance toward the human monkeypox among men who have sex with men in China

**DOI:** 10.3389/fpubh.2022.997637

**Published:** 2022-10-25

**Authors:** Min Zheng, Chenyuan Qin, Xiaohan Qian, Yongming Yao, Jue Liu, Zhi Yuan, Lin Ma, Jiacheng Fan, Rui Tao, Feng Zhou, Wenyan Chen, Zhilin Zhu, Min Liu, Guanghong Yang

**Affiliations:** ^1^Guizhou Center for Disease Control and Prevention, Guiyang, China; ^2^The Key Laboratory of Environmental Pollution Monitoring and Disease Control, Ministry of Education, School of Public Health and Health, Guizhou Medical University, Guiyang, China; ^3^Department of Epidemiology and Biostatistics, School of Public Health, Peking University, Beijing, China; ^4^Institute for Global Health and Development, Peking University, Beijing, China; ^5^National Health Commission Key Laboratory of Reproductive Health, Peking University, Beijing, China

**Keywords:** monkeypox, homosexual, bisexual, knowledge, vaccination, MSM

## Abstract

**Background:**

MSM individuals are at high risk of monkeypox infection, and judicious use of vaccines can control the outbreak. Therefore, we conducted a national cross-sectional survey to assess the vaccination willingness, associated factors, and related knowledges of monkeypox among MSM individuals in China.

**Methods:**

This anonymous cross-sectional study was conducted in China from July 1 to July 3, 2022, and electronic questionnaires were sent online to MSM individuals of specific institutions. Men, aged 18 or older, who had anal sex in the past year were recruited. Multivariable logistic regression models and univariable logistic regression models were performed in different groups of participants, including all eligible respondents, people with or without self-reported HIV infection, and people who had sex with at least one male sexual partner in last month.

**Results:**

A total of 2,618 male respondents, including 2,134 homosexuals and 484 bisexuals, were enrolled in our final analysis. Most of the respondents had a certain understanding of the source of infection, transmission route, and preventive measures, but lacked knowledge of the susceptible population, clinical manifestations, vaccination, and treatment. In total, 90.2% of all respondents were willing to receive the vaccines against monkeypox. Among people with self-reported HIV infection, the vaccination acceptance rate was 91.7%, while it was 89.7% in the rest. The main influencing factors were knowledge about monkeypox (moderate: aOR = 1.47, 95% CI: 1.04–2.08; high: aOR = 2.03, 95% CI: 1.23–3.34), knowledge about prevention measures (moderate: aOR = 3.52, 95% CI: 2.51–4.94; high: aOR = 5.32, 95% CI: 2.98–9.47), concerns about their susceptibility to monkeypox infection (aOR = 4.37, 95% CI: 3.29–5.80), and possible contact with people and animals in epidemic areas (aOR = 0.42, 95% CI: 0.25–0.70). For self-reported HIV-infected individuals, education (bachelor degree: aOR = 0.40, 95% CI: 0.18–0.89) and poor condom use (sometimes: aOR = 2.18, 95% CI: 1.06–4.47) may also affect the vaccination.

**Conclusions:**

There was still a lack of knowledge about the human monkeypox among MSM individuals in China. The vaccination acceptance rate of this high-risk population was high, and it was closely related to the knowledge factors, fear of infection, and possible contact with people or animals in affected areas. Targeted publicity and education of the high-risk groups, vaccination pre-arranged planning should be formulated to cope with the further development of this infectious disease.

## Introduction

Human monkeypox, a sporadic zoonosis caused by monkeypox virus (MPXV) infection, is gradually becoming the orthpoxvirus with the greatest impact on public health (1, 2). MPXV has two distinct branches of genetic evolution, namely the central African branch and west African branch. Its human infection was first detected in 1970 in a 9-year-old boy in the Democratic Republic of Congo, with rising frequency in recent years (3). For a long time, cases have been concentrated almost exclusively in rural rainforest villages of western and central Africa, and they were only occasionally imported to non-epidemic countries (1, 4–6). Infected animals and people are the main sources of infection (2). Monkeypox causes symptoms very similar to smallpox, but not as severe (3), including fever, severe headache, enlargement of lymph nodes, back pain, muscle soreness and weakness (2). The rash usually appears after 1–3 days of fever, concentrating on the face and limbs. When a rash appears, the patient is contagious (2). MPXV spreads from person to person through contact with bodily fluids, lesions on the skin or internal mucosal surfaces, contaminated objects, and respiratory droplets of an infected person (2, 3, 7). Also, multiple sources of evidence suggested that sex, especially among men who have sex with men (MSM) individuals, is a likely route of transmission (8–10). The fatality rate of west Africa branch was 3.6% (3, 7), albeit to 10.6% for central African branch that was considered to be more contagious (3). Generally, the overall case fatality rate is around 1–10%, but the risk of severe disease and death is higher in immunocompromised people (7, 8).

As of September 7, 2022, World Health Organization (WHO) has reported 54,709 laboratory-confirmed cases of monkeypox and 18 deaths from more than 100 countries/territories/territories (8). This is the first time that monkeypox has been reported locally in newly-affected countries that have no epidemiological links to countries in West or Central Africa (7, 8). On September 6, 2022, Hong Kong has announced the first case of monkeypox imported from the Philippines (8). Many cases in newly-affected areas were not presenting with the classically described clinical signs, and developed widespread rash on the body directly (8, 11). The 2022 multiple-country monkeypox outbreak was affected by complex factors, one of the major causes is stronger sexual transmission (3). To effectively use strong public health measures to prevent onward spread of the disease, judicious use of vaccines can support this response. Among cases with reported sexual orientation, 60% (1,214/2,025) identified as gay, bisexual, and other MSM individuals, and 41% (335/827) of cases with known HIV status were positive for HIV (8). Undoubtedly, MSM individuals are at high risk of monkeypox infection. Because of cross-immunization, smallpox vaccination can also be used to prevent human MPVX infection (12). In addition, a non-replicative monkeypox vaccine, JYNNEOS or IMVANEX or IMVAMUNE (MVA-BN), was approved in the United States in 2019 for people at risk of occupational exposure (13). JYNNEOS was also considered safe for HIV-infected patients (13, 14). The UK Health And Safety Agency (UKHSA) said it had obtained 20,000 doses of IMVANEX and was starting to vaccinate high-risk MSM individuals (15).

Given the large population base and absolute number of MSM population in China, it is of great prospective theoretical and practical significance to investigate the monkeypox related knowledge level and vaccination willingness in this high-risk population in advance (16). Therefore, we conducted a timely national cross-sectional study to assess the vaccination willingness and related knowledges among MSM individuals in China, during monkeypox outbreaks in countries other than Africa, to provide scientific basis for the prevention and control of monkeypox in the next step.

## Methods

### Study design, population, and sampling

This anonymous cross-sectional study was conducted in China from July 1 to July 3, 2022, *via* an electronic questionnaire distribution platform called Wen Juan Xing (Changsha Ranxing Information Technology Co., Ltd., Hunan, China). Considering the particularity of the study population and expert advices, we adopted the convenience sampling method to recruit a sample population. Electronic questionnaires were distributed *via* the Internet to specific MSM individuals in pre-existing organizations of homosexuals and bisexuals. Recruitment criteria are as follows: (1) understanding the study purpose and being willing to fill in the questionnaire carefully; (2) ≥18 years old; (3) men who have had anal sex in the past year.

### Questionnaire design

A self-administered questionnaire was designed to collect information from the MSM individuals, including sociodemographic characteristics, history of sexually transmitted diseases, characteristics related to sexual activity, monkeypox related cognition and their attitude toward the vaccines. All items were stated by a panel of experts, including one public health expert and two epidemiologists specializing in infectious diseases. Effective quality control was carried out through questionnaire filling limitation. Invalid questionnaires, whose respondents were female or did not meet the requirements of sexual orientation, were manually eliminated. A pilot survey involving 20 MSM individuals was conducted before it was officially released.

Acceptance rate toward vaccines against monkeypox was our primary outcome, and it was defined as the proportion of participants who answered “Yes” when asked whether they were willing to receive vaccines against monkeypox if available. Sociodemographic characteristics included gender, age, province, educational level, and occupation. The history of syphilis infection and HIV infection were the two sexually transmitted diseases concerned in this study. As for the characteristics related to sexual activity, all respondents were required to report their sexual orientation, number of sexual partners in the last month, and condom use.

Seven items were set to assess people's knowledges of monkeypox and measures to prevent it. Sources of monkeypox infection, possible routes of transmission, susceptible groups, general clinical symptoms, vaccines, specific drugs, and preventive measures were all included in this section. To better quantify the results, each correct choice got 1 point. A total of 16 scores and 7 scores were assigned the abovementioned two parts, respectively. Participants were divided into “low,” “moderate,” and “high” according to their final scores of these two knowledge factors. Besides, the respondents' contact with people or animals related to the epidemic areas, whether they had similar symptoms of monkeypox infection in the past 2 months, and whether they were worried about catching this infectious disease were also the factors to be considered.

### Statistical analysis

Descriptive analyses consisting of frequencies and percentages were used to summarize the characteristics of the recruited population. The proportion and 95% CIs of participants' willingness to receive vaccines against monkeypox were calculated, with independent Chi-square test to compare the difference. To identify factors that influence vaccination willingness, univariable and multivariable logistic regression models were performed in different groups of participants, including all eligible respondents, people with or without self-reported HIV infection, and people who had sex with at least one male sexual partner in last month. Through a backward stepwise method (*P* < 0.2), crude odds ratios (cORs) and adjusted odds ratios (aORs) were calculated, presenting with 95% confidence intervals (CIs) and *P*-values. Hosmer and Lemeshow Test was used to assess the goodness of model fitting. All data analyses in this study were conducted by SPSS 26.0 (IBM SPSS Inc., New York, USA), and two-sided *P* < 0.05 was considered statistically significant.

## Results

### Study sample characteristics

A total of 2,618 male respondents from 28 provinces, including 2,134 homosexuals and 484 bisexuals, were enrolled in our final analysis (Supplementary Figure S1, Table 1). Among them, more than half were younger than 30 years old (50.7%), lived in eastern China (52.1%), and at least had a bachelor's degree (50.9%). In terms of the history of sexually transmitted diseases, 393 (15.0%) and 722 (27.6%) were with self-reported syphilis and HIV infection, respectively. 55.5% of them had sexual intercourse with male in the past month, and 196 (7.5%) had three or more male sexual partners. However, the results showed that only 68.6% of these participants used condoms every time they had sex. For monkeypox related cognition, most people have a moderate or higher understanding of monkeypox and its prevention, and 75.0% showed their concerns about their susceptibility to monkeypox infection.

**Table 1:** Basic characteristics and acceptance toward the vaccines against human monkeypox among the Chinese MSM individuals.

**Characteristics**	**Total (*****n*** = **2,618)**				**People with HIV (*****n*** = **722)**			
	**Number (%)**	**Vaccination willingness**		***P*-value**	**Number (%)**	**Vaccination willingness**		***P*-value**
		**Yes (%)**	**95% CI**			**Yes (%)**	**95% CI**	
Total	2,618 (100)	2,362 (90.2)	(89.0–91.3)		722 (100)	662 (91.7)	(89.5–93.5)	
**Sociodemographic characteristics**								
**Region**								
Eastern	1,365 (52.1)	1,244 (91.1)	(89.5–92.6)	0.245	375 (51.9)	346 (92.3)	(89.2–94.6)	0.843
Central	601 (23.0)	538 (89.5)	(86.9–91.8)		178 (24.7)	162 (91.0)	(86.1–94.6)	
Western	652 (24.9)	580 (89.0)	(86.4–91.2)		169 (23.4)	154 (91.1)	(86.1–94.7)	
**Age group (years)**								
< 25	703 (26.9)	638 (90.8)	(88.4–92.7)	0.468	117 (16.2)	106 (90.6)	(84.3–94.9)	0.479
25–29	624 (23.8)	552 (88.5)	(85.8–90.8)		169 (23.4)	150 (88.8)	(83.3–92.9)	
30–34	548 (20.9)	497 (90.7)	(88.0–92.9)		183 (25.3)	171 (93.4)	(89.2–96.4)	
35–39	346 (13.2)	311 (89.9)	(86.4–92.7)		127 (17.6)	117 (92.1)	(86.5–95.9)	
≥40	397 (15.2)	364 (91.7)	(88.7–94.1)		126 (17.5)	118 (93.7)	(88.4–97.0)	
**Education**								
High school and below	564 (21.5)	510 (90.4)	(87.8–92.6)	0.695	214 (29.6)	199 (93.0)	(89.0–95.8)	0.365
Junior college degree	723 (27.6)	659 (91.1)	(88.9–93.1)		197 (27.3)	184 (93.4)	(89.3–96.3)	
Bachelor degree	1,060 (40.5)	952 (89.8)	(87.9–91.5)		262 (36.3)	234 (89.3)	(85.1–92.6)	
Master degree or above	271 (10.4)	241 (88.9)	(84.8–92.2)		49 (6.8)	45 (91.8)	(81.8–97.2)	
**Occupation**								
Students	492 (18.8)	441 (89.6)	(86.7–92.1)	0.307	58 (8.0)	53 (91.4)	(82.1–96.6)	0.927
Employees of enterprises and public institutions	760 (29.0)	684 (90.0)	(87.7–92.0)		198 (27.4)	181 (91.4)	(86.9–94.7)	
Workers	274 (10.5)	251 (91.6)	(87.9–94.5)		81 (11.2)	76 (93.8)	(87.0–97.6)	
Self-employed entrepreneur	214 (8.2)	198 (92.5)	(88.4–95.5)		55 (7.6)	50 (90.9)	(81.2–96.4)	
Commercial sex workers	402 (15.4)	370 (92.0)	(89.1–94.4)		152 (21.1)	142 (93.4)	(88.6–96.6)	
Unemployed	222 (8.5)	197 (88.7)	(84.1–92.4)		89 (12.3)	80 (89.9)	(82.4–94.9)	
Others	254 (9.7)	221 (87.0)	(82.5–90.7)		89 (12.3)	80 (89.9)	(82.4–94.9)	
**History of sexually transmitted diseases**								
**Syphilis infection**								
Yes	393 (15.0)	356 (90.6)	(87.4–93.2)	0.001^*^	262 (36.3)	237 (90.5)	(86.5–93.6)	0.544
No	2,132 (81.4)	1,933 (90.7)	(89.4–91.8)		443 (61.4)	410 (92.6)	(89.8–94.7)	
Not sure	93 (3.6)	73 (78.5)	(69.4–85.9)		17 (2.4)	15 (88.2)	(67.3–97.5)	
**HIV infection**								
Yes	722 (27.6)	662 (91.7)	(89.5–93.5)	0.004^*^	–	–	–	–
No	1,760 (67.2)	1,588 (90.2)	(88.8–91.5)		–	–	–	
Not sure	136 (5.2)	112 (82.4)	(75.3–88.0)		–	–	–	
**Characteristics related to sexual activity**								
**Sexual orientation**								
Homosexual	2,134 (81.5)	1,931 (90.5)	(89.2–91.7)	0.336	613 (84.9)	559 (91.2)	(88.8–93.2)	0.249
Bisexual	484 (18.5)	431 (89.0)	(86.0–91.6)		109 (15.1)	103 (94.5)	(89.0–97.7)	
**Number of sexual partners in the last month**								
0	1,165 (44.5)	1,041 (89.4)	(87.5–91.0)	0.167	358 (49.6)	327 (91.3)	(88.1–93.9)	0.892
1–2	1,257 (48.0)	1,148 (91.3)	(89.7–92.8)		321 (44.5)	296 (92.2)	(88.9–94.8)	
≥3	196 (7.5)	173 (88.3)	(83.2–92.2)		43 (6.0)	39 (90.7)	(79.4–96.8)	
**Condom use**								
Never	110 (4.2)	91 (82.7)	(74.9–88.9)	0.024^*^	28 (3.9)	24 (85.7)	(69.5–95.0)	0.383
Sometimes	712 (27.2)	647 (90.9)	(88.6–92.8)		217 (30.1)	202 (93.1)	(89.1–95.9)	
Every time	1,796 (68.6)	1,624 (90.4)	(89.0–91.7)		477 (66.1)	436 (91.4)	(88.6–93.7)	
**Monkeypox related cognition**								
**Knowledges of monkeypox**								
Low (scores 0–5)	827 (31.6)	668 (80.8)	(78.0–83.3)	< 0.001^*^	238 (33.0)	203 (85.3)	(80.4–89.4)	< 0.001^*^
Moderate (scores 6–11)	1,007 (38.5)	938 (93.1)	(91.5–94.6)		261 (36.1)	242 (92.7)	(89.1–95.4)	
High (scores12–16)	784 (29.9)	756 (96.4)	(95.0–97.6)		223 (30.9)	217 (97.3)	(94.5–98.9)	
**Knowledges of preventing monkeypox**								
Low (scores 0–2)	704 (26.9)	536 (76.1)	(72.9–79.2)	< 0.001^*^	198 (27.4)	155 (78.3)	(72.2–83.6)	< 0.001^*^
Moderate (scores 3–5)	1,256 (48.0)	1,186 (94.4)	(93.1–95.6)		343 (47.5)	327 (95.3)	(92.7–97.2)	
High (scores 6–7)	658 (25.1)	640 (97.3)	(95.8–98.3)		181 (25.1)	180 (99.4)	(97.4–99.9)	
**Contact with people and animals in epidemic area**								
No	2,485 (94.9)	2,253 (90.7)	(89.5–91.8)	0.001^*^	694 (96.1)	639 (92.1)	(89.9–93.9)	0.062
Yes	133 (5.1)	109 (82.0)	(74.8–87.8)		28 (3.9)	23 (82.1)	(65.2–92.8)	
**Similar symptoms in last 2 months** ^**§**^								
No	2,448 (93.5)	2,213 (90.4)	(89.2–91.5)	0.243	664 (92.0)	609 (91.7)	(89.4–93.6)	0.929
Yes	170 (6.5)	149 (87.6)	(82.1–91.9)		58 (8.0)	53 (91.4)	(82.1–96.6)	
**Perceived susceptibility** ^**¶**^								
Yes	1,964 (75.0)	1,864 (94.9)	(93.9–95.8)	< 0.001^*^	570 (78.9)	544 (95.4)	(93.5–96.9)	< 0.001^*^
No	410 (15.7)	361 (88.0)	(84.6–90.9)		89 (12.3)	76 (85.4)	(77.0–91.6)	
Not sure	244 (9.3)	137 (56.1)	(49.9–62.3)		63 (8.7)	42 (66.7)	(54.5–77.4)	

* P < 0.05.

§ Six main symptoms of monkeypox were investigated in this questionnaire, including chills and fever, enlarged lymph nodes, single or multiple rashes on the genitals or perianal area or other parts of the body, weak, muscle pain and headache. Having any of these symptoms in the last 2 months was defined as “Yes”.

¶ All respondents were asked to answer the question “Are you worried about infecting monkeypox?” Answers included “Yes,” “No,” and “Not sure”.

Stratified by HIV infection, 722 (27.6%) respondents self-reported their infections of HIV (Table 1). Among them, 364 (50.4%) had ≥1 male sexual partners in the last month, and 245 (33.9%) never or only sometimes used condoms when having sex. 28 (3.9%) of them reported a possible contact with people or animals in epidemic area. 58 (8.0%) participants reported at least one susceptible symptom associated with monkeypox. Additionally, 78.9% have perceived their susceptibility to monkeypox.

### Participants' knowledge of monkeypox and its prevention measures

For all respondents, 82.8% (2,167/2,618) of them agreed that monkeypox patients were the source of infection, while 13.8% of participants knew nothing about it (Table 2). Close contact with infected persons (78.9%), sexual transmission (64.4%) and contact with contaminated materials (61.0%) were the three main routes of transmission they agreed. More than half (53.8%) believed that all people were susceptible, and only 40.2 and 33.3% of participants thought MSM individuals and HIV infected persons were high-risk groups. Only 23.0% knew that monkeypox could be prevented by vaccines. And 62.8% of them were aware of the benefits of reducing the number of sexual partners and continuing condom use to prevent infection.

**Table 2:** Participants' knowledges of monkeypox and its prevention measures.

**Items**	**Total**	**People with HIV**	**At least one sexual partner^§§^**
	**(*n* = 2,618)**	**(*n* = 722)**	**(*n* = 1,453)**
	***N* (%)**	***N* (%)**	***N* (%)**
**Q1: What are the sources of monkeypox? (2 points)**			
Infected animals	1,899 (72.5)	525 (72.7)	1,053 (72.5)
Infected people	2,167 (82.8)	585 (81.0)	1,196 (82.3)
Unclear	348 (13.3)	106 (14.7)	192 (13.2)
**Q2: What are the possible transmission routes of monkeypox? (5 points)**			
Close contact with patients	2,066 (78.9)	558 (77.3)	1,146 (78.9)
Droplet transmission	1,244 (47.5)	328 (45.4)	678 (46.7)
Contact with contaminated items	1,596 (61.0)	451 (62.5)	876 (60.3)
Vertical transmission	1,012 (38.7)	287 (39.8)	570 (39.2)
Sexual transmission	1,685 (64.4)	470 (65.1)	942 (64.8)
Unclear	393 (15.0)	112 (15.5)	210 (14.5)
**Q3: What are the people susceptible to monkeypox? (3 points)**			
All	1,405 (53.7)	385 (53.3)	770 (53.0)
People who were not vaccinated against smallpox	1,193 (45.6)	337 (46.7)	670 (46.1)
MSM individuals	1,092 (41.7)	329 (45.6)	638 (43.9)
People with self-reported HIV infection	909 (34.7)	278 (38.5)	520 (35.8)
Unclear	409 (15.6)	112 (15.5)	215 (14.8)
**Q4: What are the common clinical symptoms of monkeypox? (6 points)**			
Chills and fever	1,280 (48.9)	336 (46.5)	730 (50.2)
Enlarged lymph nodes	1,239 (47.3)	336 (46.5)	699 (48.1)
Single or multiple rashes on the genitals or perianal area or other parts of the body	1,409 (53.8)	380 (52.6)	791 (54.4)
Weak	984 (37.6)	251 (34.8)	558 (38.4)
Muscle pain	1,051 (40.1)	283 (39.2)	596 (41.0)
Headache	910 (34.8)	247 (34.2)	522 (35.9)
Unclear	942 (36.0)	284 (39.3)	516 (35.3)
**Q5: Is it possible to prevent monkeypox infection by vaccination? (1 point)**			
No	970 (37.1)	285 (39.5)	535 (36.8)
Yes	601 (23.0)	151 (20.9)	336 (23.1)
Unclear	1,047 (40.0)	286 (39.6)	582 (40.1)
**Q6: Is there any specific medicine that can treat monkeypox? (1 point)**			
Yes	149 (5.7)	34 (4.7)	83 (5.7)
No	1,315 (50.2)	371 (51.4)	735 (50.6)
Unclear	1,154 (44.1)	317 (43.9)	635 (43.7)
**Q7: What can be done to prevent monkeypox infection? (5 points)**			
Stay away from the outbreak areas	2,004 (76.5)	546 (75.6)	1,118 (76.9)
Vaccination	1,350 (51.6)	359 (49.7)	764 (52.6)
Wearing mask	1,500 (57.3)	406 (56.2)	818 (56.3)
Hand disinfection	1,662 (63.5)	458 (63.4)	919 (63.2)
Regular sex partner and condom use	1,643 (62.8)	459 (63.5)	918 (63.2)
Unclear	324 (12.4)	95 (13.1)	168 (11.6)

§§ This group was defined as having had sex with at least one male partner in the past month (MSM).

For people at least had one sexual partner in last month, nearly a third of MSM individuals were still unaware of the role of sexual behavior in transmission process, and only 43.9% (638/1,453) realized their own risk of infection (Table 2). As it shown in Supplementary Table S1, Table 2, most of the respondents (four groups) had a certain degree of understanding of the source of infection, transmission route, and preventive measures, but lacked knowledge of the susceptible population, clinical manifestations, vaccination, and treatment. Knowledges of monkeypox among the three subgroups was like those in total population.

### Vaccination acceptance toward the monkeypox among Chinese homosexual and bisexual adults

In total, 90.2% (2,362/2,618) of all respondents were willing to receive the vaccines against monkeypox (Table 1). The vaccine acceptance rates among people with self-reported syphilis infection, non-consistent condom use, and those who thought they were at risk were 90.6, 90.9, and 94.9%, respectively. Surprisingly, those who reported contact with people or animals from the epidemic areas were less likely to be vaccinated than those who had not. Among people with self-reported HIV infection, the vaccination acceptance rate was 91.7% (662/722), while 89.7% (1,700/1,896) in the rest population. Similar increases in vaccination acceptance were seen in subgroups as knowledge scores increased.

### Factors related to the acceptance toward the vaccines against monkeypox

Multivariable models showed that, for all eligible participants, moderate and high levels of knowledge about monkeypox (moderate: aOR = 1.47, 95% CI: 1.04–2.08; high: aOR = 2.03, 95% CI: 1.23–3.34) and its prevention measures (moderate: aOR = 3.52, 95% CI: 2.51–4.94; high: aOR = 5.32, 95% CI: 2.98–9.47) were associated with high acceptance toward the monkeypox vaccines (Table 3). People who showed their concerns about their susceptibility to monkeypox infection (aOR = 4.37, 95% CI: 3.29–5.80) were more likely to receive the vaccines. However, possible contact with people and animals in epidemic areas exhibited an unwillingness to getting vaccinated. For people with self-reported HIV, knowledge level on prevention measures (moderate: aOR = 7.18, 95% CI: 3.62–14.23) and perceived susceptibility (aOR = 5.37, 95% CI: 2.90–9.94) were also the factors influenced participants' vaccination willingness. In contrast, The higher the education level, the lower the vaccination acceptance (bachelor degree: aOR = 0.40, 95% CI: 0.18–0.89). Compared to people who used condoms consistently, other people were more likely to be vaccinated (sometimes: aOR = 2.18, 95% CI: 1.06–4.47). The results of people without HIV were like those in all participants. Univariable logistic regression models of all participants, people with self-reported HIV infection and those without HIV infection were shown in Supplementary Table S2.

**Table 3:** Multivariable logistic regression of the factors associated with vaccine acceptance among the Chinese MSM individuals^**^.

**Characteristics**	**Total (*****n*** = **2,618)**		**People with HIV (*****n*** = **722)**		**People without HIV**^**†**^ **(*****n*** = **1,896)**	
	**aOR (95% CI)**	***P*-value**	**aOR (95% CI)**	***P*-value**	**aOR (95% CI)**	***P*-value**
**Education**						
High school and below	–	–	1 (reference)		–	–
Junior college degree	–	–	0.68 (0.28–1.66)	0.399	–	–
Bachelor degree	–	–	0.40 (0.18–0.89)	0.024^*^	–	–
Master degree or above	–	–	0.44 (0.12–1.63)	0.220	–	–
**Condom use**						
Never	–	–	0.64 (0.18–2.24)	0.483	–	–
Sometimes	–	–	2.18 (1.06–4.47)	0.034^*^	–	–
Every time	–	–	1 (reference)		–	–
**Knowledges of monkeypox**						
Low	1 (reference)		–	–	1 (reference)	
Moderate	1.47 (1.04–2.08)	0.030^*^	–	–	1.82 (1.21–2.72)	< 0.001^*^
High	2.03 (1.23–3.34)	0.005^*^	–	–	2.42 (1.36–4.29)	< 0.001^*^
**Knowledges of preventing monkeypox**						
Low	1 (reference)		1 (reference)		1 (reference)	
Moderate	3.52 (2.51–4.94)	< 0.001^*^	7.18 (3.62–14.23)	< 0.001^*^	3.05 (2.06–4.52)	< 0.001^*^
High	5.32 (2.98–9.47)	< 0.001^*^	58.34 (7.65–445.01)	< 0.001^*^	3.60 (1.92–6.75)	< 0.001^*^
**Contact with people and animals in epidemic area**						
Yes	0.42 (0.25–0.70)	0.001^*^	0.36 (0.11–1.24)	0.106	0.43 (0.24–0.76)	0.004^*^
No	1 (reference)		1 (reference)		1 (reference)	
**Perceived susceptibility** ^¶^						
Yes	4.37 (3.29–5.80)	< 0.001^*^	5.37 (2.90–9.94)	< 0.001^*^	4.17 (3.00–5.79)	< 0.001^*^
No or not sure	1 (reference)		1 (reference)		1 (reference)	

* P < 0.05.

† This group includes people who have tested negative for HIV and those who have not been tested.

¶ All respondents were asked to answer the question “Are you worried about infecting monkeypox?” Answers include “Yes,” “No,” and “Not sure.”

** Only statistically significant results were retained. Complete results are shown in Supplementary Table S3.

**Figure 1:**
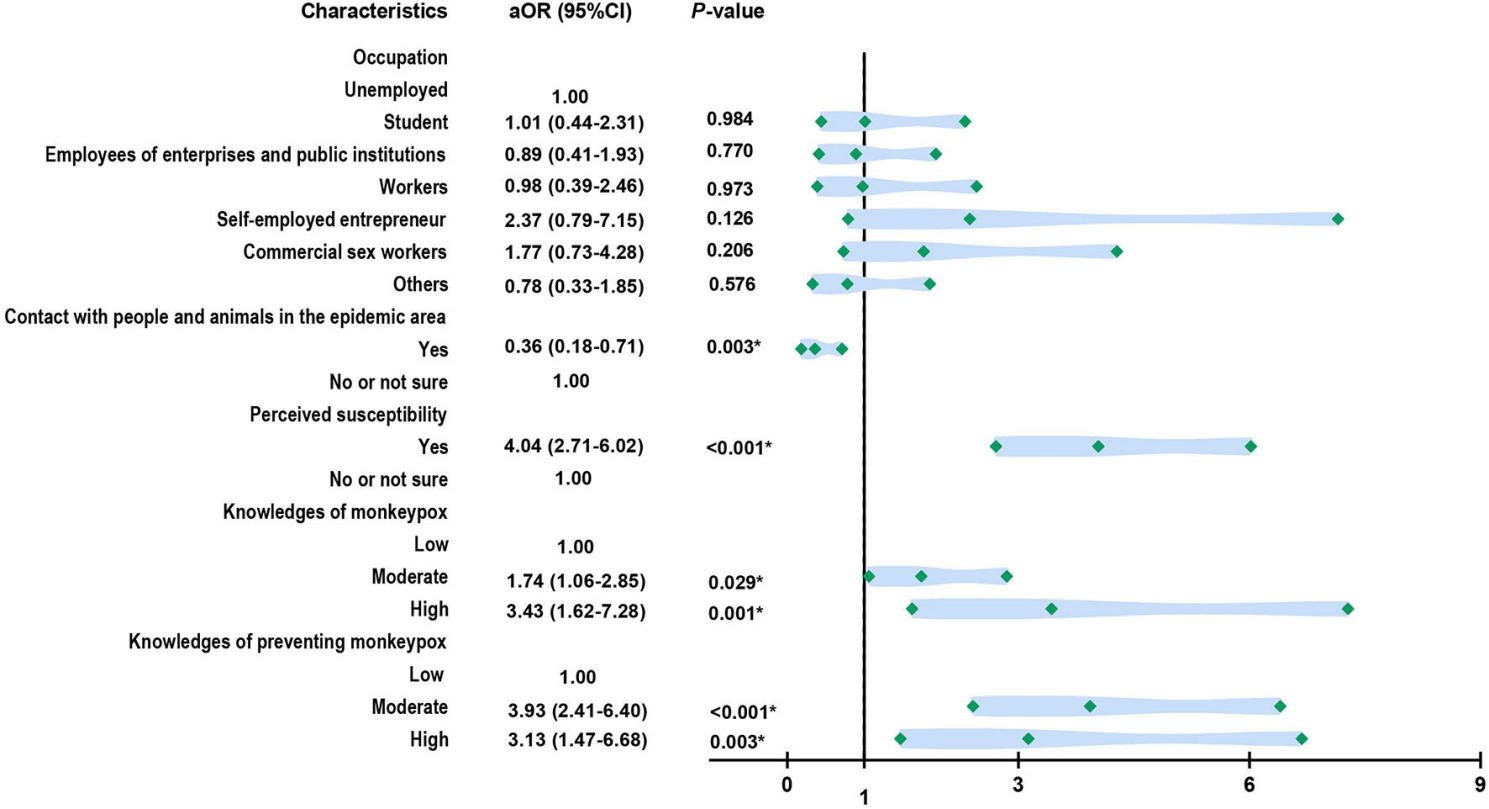
Factors associated with vaccine acceptance among the Chinese MSM individuals who had at least one sex partner last month (*n* = 1,453).

Additionally, for 1,453 people who had sex intercourse with at least one sexual partner in last month, 90.9% of them showed willingness to be vaccinated. Similarly, perceived susceptibility (aOR = 4.04, 95% CI: 2.71–6.02), more knowledges of monkeypox (moderate: aOR = 1.74, 95% CI: 1.06–2.85; high: aOR = 3.43, 95% CI: 1.62–7.28), and more knowledges of preventing this infectious disease (moderate: aOR = 3.93, 95% CI: 2.41–6.40; high: aOR = 3.13, 95% CI: 1.47–6.68) were positive factors that could promote people's vaccination acceptance (Figure 1). Participants who encountered people or animals in epidemic areas were less likely to get vaccinated (aOR = 0.36, 95% CI: 0.18–0.71).

## Discussion

Evidently, this is first study targeting MSM individuals to assess their knowledge of monkeypox, and to explore the vaccination acceptance and factors that may influence their willingness. Our results demonstrated that most of the respondents had a certain degree of understanding of the source of infection, transmission route, and preventive measures, but lacked knowledge of the susceptible population, clinical manifestations, vaccination, and treatment. In total, 90.2% of all respondents were willing to receive the vaccines against monkeypox. Knowledge about monkeypox and its prevention measures, perceived susceptibility, and possible contact with people and animals in epidemic areas were the main associated factors. For people with self-reported HIV infection, poor condom use had a positive effect on vaccination intentions, while higher education did have a negative one. At present, the multiple-country monkeypox outbreak has constitute a Public Health Emergency of International Concern (2). Therefore, clarifying the risk awareness and vaccination willingness of MSM population for monkeypox infection in advance is helpful to carry out targeted knowledge popularization and develop vaccination plans to deal with the further development of the epidemic.

Among these four groups, most of the respondents had a certain degree of understanding of human monkeypox, but lacked knowledge of the susceptible population, clinical manifestations, and treatment. In our study, few people who knew that HIV-infected people were susceptible, while more than 25% of participants self-reported HIV infection. Thus, greater attention should be paid to HIV-infected people in MSM population because of the higher risk of severe disease and death in the immunocompromised population (8). In addition, about 40% of respondents knew nothing about the clinical manifestations of MPXV infection, and the rest only had poor awareness of specific symptoms. Actually, studies have shown that monkeypox patients in 2022 tend to have atypical symptoms and may develop flu-like symptoms before the rash begins, which is not conducive to self-health monitoring in key populations (7, 8). As for vaccines against monkeypox, 77% had an incorrect opinion about vaccines or did not know whether it could be prevented by vaccines. Evidently, to better control the epidemic of monkeypox, one of the current priorities is to raise MSM and HIV-infected population's awareness of the risk of MPVX infection, self-health monitoring, scientific prevention, and prompt treatment of suspicious symptoms.

Under the current circumstances, MSM populations show a high willingness to be vaccinated, and the multiple-country monkeypox outbreak has constituted a Public Health Emergency of International Concern now (2). According to our research, 90.2% of all respondents were willing to receive the vaccines against monkeypox. For people with self-reported HIV infection, the acceptance rate was 91.7%, while 90.9% for people who had sex intercourse with at least one sexual partner in last month. Currently, there is no specific approved vaccine in the clinic for MPXV. However, vaccination against the smallpox virus provided cross-protection against MPXV based on the orthpoxvirus immune response (12). However, both the first- and second-generation vaccines contain replicable vaccinia viruses, which pose a risk of adverse events, especially in populations with varying degrees of immunosuppression (17–19). JYNNEOS (IMVANEX or IMVAMUNE) is a third-generation smallpox vaccine that has been shown to have good safety and immunogenicity in HIV-infected patients and patients with atopic dermatitis (14, 20–22). It was approved to prevent monkeypox infection in the United States in 2019 (3, 13). At a recent meeting, the UKHSA said 20,000 doses of IMVANEX vaccine had been distributed to clinics where MSM individuals will be vaccinated (15). Countries such as Canada, The UK and the US have also started “ring vaccination” strategy, vaccinating people in close contact with infected person (23).

We found that, the higher the MSM population's knowledges of monkeypox (e.g., sources of infection, transmission routes, susceptible groups, clinical manifestations, prevention, and treatment measures), the higher the acceptance of vaccination acceptance. Previous studies supported those higher levels of knowledge on epidemiological characteristics, the number of deaths, and effective prevention measures of infectious diseases were associated with positive behavior change (24, 25). At different stages of an epidemic, good awareness of this infectious disease and vaccines boosted people's willingness to be vaccinated in China, Brazil, Malaysia, Singapore, Colombia, and other countries (26–29). Perceptions or beliefs about outbreaks are important in deciding on specific preventive actions (30, 31). Some studies suggested that the fear of infection may also play a role in motivating people to get vaccinated, which is consistent with our results (32–34). But surprisingly, compared to people without the history of epidemiology, people who had contact with people or animals in epidemic areas were less likely to be vaccinated. On the one hand, the results may not be universal due to the insufficient sample size included in this study. On the other hand, more thematic studies may be needed to further investigate the specific reasons, as this group of people should be the focus of infectious disease prevention and control. For people with self-reported HIV infection, higher education level may make them more cautious about vaccines, and they may avoid vaccination behaviors in non-emergency situations (35). Besides, people who did not consistently use condoms had a higher willingness to be vaccinated, which was related to their higher perceived susceptibility (32–34).

As the first survey to explore MSM individuals' knowledge of monkeypox, vaccination willingness and influencing factors among MSM individuals, our study provided basic data for protecting high-risk groups and responding to the further development of this epidemic. However, there were several limitations in our study. First, given the particularity of the study population (homosexuals and bisexuals), this survey adopted convenience sampling method, and recruited research subjects from MSM population organizations through issuing electronic questionnaire on the Internet. This method may be less accurate and representative than probabilistic sampling. Second, people's attitude toward vaccines against monkeypox was measured only by this self-reported questionnaire, and we were unable to assess it *via* standard scales. Meanwhile, no standard scales were established to evaluate participants' knowledge of monkeypox and related preventive measures. To a certain extent, it is inevitable for participants to search for answers on the Internet, although we have set up answer reminders, text that cannot be copied and pasted directly, and limited answer time. Third, self-reported data, some of which is sensitive (e.g., sexual behavior, history of sexually transmitted diseases), may not always be accurately reported. Our study is only a rough supplement to existing research gap, and it is unclear whether vaccination will be carried out in the Chinese MSM population. We did not study the specific selection of vaccines (ACAC2000 or JYNNEOS) and the knowledge about the side effects of the vaccine and its effects on the choice of the vaccination. Of course, in order to respond to this infectious disease in advance, we also hope to carry out a large-scale offline survey with more participants as soon as possible, so as to obtain more accurate and reliable data.

## Conclusions

In conclusion, MSM people in China have paid high attention to human monkeypox, but there was still a lack of knowledge about the susceptible groups, clinical symptoms, and preventive measures. The vaccination acceptance rate of this high-risk population was high, and it was related to the knowledge factors, fear of infection, and possible contact with people or animals in affected areas. Education and condom use may also influence the vaccination behavior among self-reported HIV-infected individuals. More attention should be paid to the publicity and education of the public and high-risk groups (such as MSM individuals and HIV infected people), surveillance of MPVX infection in key population, and removing the barriers to vaccination. Targeted vaccination pre-arranged planning is supposed to be formulated to cope with the further development of human monkeypox in China.

## Data availability statement

The original contributions presented in the study are included in the article/Supplementary material, further inquiries can be directed to the corresponding author/s.

## Ethics statement

The studies involving human participants were reviewed and approved by the Ethics Committee of Guizhou Center for Disease Control and Prevention (Q2022-02). The patients/participants provided their written informed consent to participate in this study.

## Author contributions

CQ, WC, ZZ, and MZ: conceptualization. CQ and MZ: methodology and analysis and writing-original draft preparation. CQ: visualization. XQ, YY, ZY, LM, JF, RT, FZ, and ML: review and editing. JL and GY: supervision. All authors have read and agreed to the published version of the manuscript.

## Funding

This study was funded by the Science and Technology Support Project of Guizhou Science and Technology Department ([2021]026), the National Natural Science Foundation of China (72122001 and 71934002), the National Statistical Science Research Project (2021LY038), the National R&D Key Project (2021ZD0114101, 2021ZD0114104, and 2021ZD0114105), and the National Science and Technology Project on Development Assistance for Technology, Developing China-ASEAN Public Health Research and Development Collaborating Center (KY202101004), and the Fundamental Research Funds for the Central Universities supported by Global Center for Infectious Disease and Policy Research and Global Health and Infectious Diseases Group, of Peking University (202204).

## Conflict of interest

The authors declare that the research was conducted in the absence of any commercial or financial relationships that could be construed as a potential conflict of interest.

## Publisher's note

All claims expressed in this article are solely those of the authors and do not necessarily represent those of their affiliated organizations, or those of the publisher, the editors and the reviewers. Any product that may be evaluated in this article, or claim that may be made by its manufacturer, is not guaranteed or endorsed by the publisher.
