# Supplementary material for: Knowledge and vaccination acceptance toward the human monkeypox among men who have sex with men in China
Source: Front Public Health. 2022 Oct 25;10:997637. doi: 10.3389/fpubh.2022.997637 (PMC9640956; doi:10.3389/fpubh.2022.997637)
Supplement: Supplementary file 1 [file Data_Sheet_1.docx]

| **Table S1** Scores of knowledges about monkeypox and its prevention measures | | | | | | | | | | | |
| --- | --- | --- | --- | --- | --- | --- | --- | --- | --- | --- | --- |
| **Questions** | **Total (n=2,618)** | |  | **People with HIV (n=722)** | |  | **People without HIV^†^ (n=1,896)** | |  | **At least one sexual partner**^§§^ **(n=1,453)** | |
|  | N (%) | Average scores |  | N (%) | Average scores |  | N (%) | Average scores |  | N (%) | Average scores |
| **Q1: What are the sources of monkeypox? (2 points)** | | | | | | | | | | | |
|  |  | 1.55 |  |  | 1.54 |  |  | 1.56 |  |  | 1.55 |
| 0 | 348 (13.3%) |  |  | 106 (14.7%) |  |  | 242 (12.8%) |  |  | 192 (13.2%) |  |
| 1 | 474 (18.1%) |  |  | 122 (16.9%) |  |  | 352 (18.6%) |  |  | 273 (18.8%) |  |
| 2 | 1796 (68.6%) |  |  | 494 (68.4%) |  |  | 1302 (68.7%) |  |  | 988 (68.0%) |  |
| **Q2: What are the possible transmission routes of monkeypox? (5 points)** | | | | | | | | | | | |
|  |  | 2.90 |  |  | 2.90 |  |  | 2.91 |  |  | 2.90 |
| 0 | 393 (15.0%) |  |  | 112 (15.5%) |  |  | 281 (14.8%) |  |  | 210 (14.5%) |  |
| 1 | 218 (8.3%) |  |  | 49 (6.8%) |  |  | 169 (8.9%) |  |  | 133 (9.2%) |  |
| 2 | 385 (14.7%) |  |  | 120 (16.6%) |  |  | 265 (14.0%) |  |  | 225 (15.5%) |  |
| 3 | 530 (20.2%) |  |  | 139 (19.3%) |  |  | 391 (20.6%) |  |  | 281 (19.3%) |  |
| 4 | 435 (16.6%) |  |  | 122 (16.9%) |  |  | 313 (16.5%) |  |  | 234 (16.1%) |  |
| 5 | 657 (25.1%) |  |  | 180 (24.9%) |  |  | 477 (25.2%) |  |  | 370 (25.5%) |  |
| **Q3: What are the people susceptible to monkeypox? (3 points)** | | | | | | | | | | | |
|  |  | 1.22 |  |  | 1.31 |  |  | 1.19 |  |  | 1.26 |
| 0 | 1130 (43.2%) |  |  | 306 (42.4%) |  |  | 824 (43.5%) |  |  | 599 (41.2%) |  |
| 1 | 452 (17.3%) |  |  | 96 (13.3%) |  |  | 356 (18.8%) |  |  | 259 (17.8%) |  |
| 2 | 366 (14.0%) |  |  | 112 (15.5%) |  |  | 254 (13.4%) |  |  | 216 (14.9%) |  |
| 3 | 670 (25.6%) |  |  | 208 (28.8%) |  |  | 462 (24.4%) |  |  | 379 (26.1%) |  |
| **Q4: What are the common clinical symptoms of monkeypox? (6 points)** | | | | | | | | | | | |
|  |  | 2.63 |  |  | 2.54 |  |  | 2.65 |  |  | 2.40 |
| 0 | 942 (36.0%) |  |  | 284 (39.3%) |  |  | 658 (34.7%) |  |  | 516 (35.5%) |  |
| 1 | 213 (8.1%) |  |  | 57 (7.9%) |  |  | 156 (8.2%) |  |  | 109 (7.5%) |  |
| 2 | 224 (8.6%) |  |  | 50 (6.9%) |  |  | 174 (9.2%) |  |  | 119 (8.2%) |  |
| 3 | 254 (9.7%) |  |  | 62 (8.6%) |  |  | 192 (10.1%) |  |  | 141 (9.7%) |  |
| 4 | 176 (6.7%) |  |  | 48 (6.6%) |  |  | 128 (6.8%) |  |  | 110 (7.6%) |  |
| 5 | 108 (4.1%) |  |  | 28 (3.9%) |  |  | 80 (4.2%) |  |  | 62 (4.3%) |  |
| 6 | 701 (26.8%) |  |  | 193 (26.7%) |  |  | 506 (26.7%) |  |  | 396 (27.3%) |  |
| **Q5: Is it possible to prevent monkeypox infection by vaccination? (1 points)** | | | | | | | | | | | |
|  |  | 0.23 |  |  | 0.21 |  |  | 0.24 |  |  | 0.23 |
| 0 | 2017 (77.0%) |  |  | 571 (79.1%) |  |  | 1446 (76.3%) |  |  | 1117 (76.9%) |  |
| 1 | 601 (23.0%) |  |  | 151 (20.9%) |  |  | 450 (23.7%) |  |  | 336 (23.1%) |  |
| **Q6: Is there any specific medicine that can treat monkeypox? (1 points)** | | | | | | | | | | | |
|  |  | 0.50 |  |  | 0.51 |  |  | 0.50 |  |  | 0.51 |
| 0 | 1303 (49.8%) |  |  | 351 (48.6%) |  |  | 952 (50.2%) |  |  | 718 (49.4%) |  |
| 1 | 1315 (50.2%) |  |  | 371 (51.4%) |  |  | 944 (49.8%) |  |  | 735 (50.6%) |  |
| **Q7: What can be done to prevent monkeypox infection? (5 points)** | | | | | | | | | | | |
|  |  | 3.12 |  |  | 3.09 |  |  | 3.13 |  |  | 3.12 |
| 0 | 324 (12.4%) |  |  | 95 (13.2%) |  |  | 229 (12.1%) |  |  | 168 (11.6%) |  |
| 1 | 280 (10.7%) |  |  | 76 (10.5%) |  |  | 204 (10.8%) |  |  | 164 (11.3%) |  |
| 2 | 306 (11.7%) |  |  | 78 (10.8%) |  |  | 228 (12.0%) |  |  | 174 (12.0%) |  |
| 3 | 415 (15.9%) |  |  | 124 (17.2%) |  |  | 291 (15.3%) |  |  | 234 (16.1%) |  |
| 4 | 443 (16.9%) |  |  | 121 (16.8%) |  |  | 322 (17.0%) |  |  | 242 (16.7%) |  |
| 5 | 850 (32.5%) |  |  | 228 (31.6%) |  |  | 622 (32.8%) |  |  | 471 (32.4%) |  |

^†^ This group includes people who have tested negative for HIV and those who have not been tested.

^§§^ This group was defined as having had sex with at least one male partner in the past month (MSM)

| **Table S2** Univariable logistic regression of the factors associated with the acceptance toward the vaccines against monkeypox among the Chinese MSM individuals | | | | | | |
| --- | --- | --- | --- | --- | --- | --- |
| **Characteristics** | **Total (n=2,618)** | | **People with HIV (n=722)** | | **People without HIV**^†^ **(n=1,896)** | |
|  | Crude OR (95% CI) | ***P*** value | Crude OR (95% CI) | ***P*** value | Crude OR (95% CI) | ***P*** value |
| **Sociodemographic characteristics** | | | | | | |
| **Region** |  |  |  |  |  |  |
| Eastern | 1.28 (0.94-1.74) | 0.120 | 1.16 (0.61-2.23) | 0.651 | 1.31 (0.92-1.85) | 0.135 |
| Central | 1.06 (0.74-1.52) | 0.749 | 0.99 (0.47-2.06) | 0.971 | 1.07 (0.71-1.61) | 0.745 |
| Western | 1 (reference) |  | 1 (reference) |  | 1 (reference) |  |
| **Age group (years)** |  |  |  |  |  |  |
| <25 | 1 (reference) |  | 1 (reference) |  | 1 (reference) |  |
| 25-29 | 0.78 (0.55-1.11) | 0.172 | 0.82 (0.37-1.79) | 0.618 | 0.77 (0.52-1.15) | 0.201 |
| 30-34 | 0.99 (0.68-1.46) | 0.971 | 1.48 (0.63-3.47) | 0.369 | 0.85 (0.55-1.31) | 0.458 |
| 35-39 | 0.91 (0.59-1.40) | 0.652 | 1.21 (0.50-2.97) | 0.671 | 0.79 (0.48-1.30) | 0.351 |
| ≥40 | 1.12 (0.73-1.74) | 0.602 | 1.53 (0.59-3.95) | 0.379 | 1.00 (0.61-1.64) | 0.996 |
| **Education** |  |  |  |  |  |  |
| High school and below | 1 (reference) |  | 1 (reference) |  | 1 (reference) |  |
| Junior college degree | 1.09 (0.75-1.59) | 0.656 | 1.07 (0.49-2.30) | 0.869 | 1.17 (0.75-1.81) | 0.490 |
| Bachelor degree | 0.93 (0.66-1.32) | 0.694 | 0.63 (0.33-1.21) | 0.167 | 1.13 (0.75-1.69) | 0.568 |
| Master degree or above | 0.85 (0.53-1.36) | 0.501 | 0.85 (0.27-2.68) | 0.779 | 0.95 (0.56-1.60) | 0.835 |
| **Occupation** |  |  |  |  |  |  |
| Student | 1.10 (0.66-1.82) |  | 1.19 (0.38-3.75) | 0.764 | 1.15 (0.63-2.11) | 0.644 |
| Employees of enterprises and public institutions | 1.14 (0.71-1.84) |  | 1.20 (0.51-2.80) | 0.677 | 1.17 (0.65-2.10) | 0.609 |
| Workers | 1.38 (0.76-2.51) |  | 1.71 (0.55-5.33) | 0.355 | 1.33 (0.65-2.71) | 0.434 |
| Self-employed entrepreneur | 1.57 (0.81-3.03) |  | 1.12 (0.36-3.55) | 0.841 | 1.84 (0.82-4.12) | 0.138 |
| Commercial sex workers | 1.47 (0.85-2.55) |  | 1.60 (0.62-4.09) | 0.329 | 1.42 (0.72-2.80) | 0.316 |
| Unemployed | 1 (reference) |  | 1 (reference) |  | 1 (reference) |  |
| Others | 0.85 (0.49-1.48) |  | 1.00 (0.38-2.65) | 1.000 | 0.80 (0.41-1.58) | 0.527 |
| **History of sexually transmitted diseases** | | | | | | |
| **HIV infection** |  |  |  |  |  |  |
| Yes | 1.27 (0.94-1.72) | 0.119 |  |  |  |  |
| No or not sure | 1 (reference) |  |  |  |  |  |
| **Syphilis infection** |  |  |  |  |  |  |
| Yes | 1.05 (0.73-1.51) | 0.792 | 0.78 (0.46-1.34) | 0.366 | 1.15 (0.63-2.13) | 0.647 |
| No or not sure | 1 (reference) |  | 1 (reference) |  | 1 (reference) |  |
| **Characteristics related to sexual activity** | | | | | | |
| **Sexual orientation** |  |  |  |  |  |  |
| Homosexual | 1.17 (0.85-1.61) | 0.337 | 0.60 (0.25-1.44) | 0.254 | 1.32 (0.93-1.87) | 0.120 |
| Bisexual | 1 (reference) |  | 1 (reference) |  | 1 (reference) |  |
| **Number of sexual partners in the last month** | | | | | | |
| 0 | 1 (reference) |  | 1 (reference) |  | 1 (reference) |  |
| 1-2 | 1.25 (0.96-1.64) | 0.101 | 1.12 (0.65-1.95) | 0.681 | 1.32 (0.97-1.80) | 0.080 |
| ≥3 | 0.90 (0.56-1.44) | 0.649 | 0.92 (0.31-2.76) | 0.888 | 0.92 (0.54-1.56) | 0.752 |
| **Condom use** |  |  |  |  |  |  |
| Never | 0.51 (0.30-0.85) | 0.010* | 0.56 (0.19-1.70) | 0.310 | 0.49 (0.27-0.89) | 0.018* |
| Sometimes | 1.05 (0.78-1.42) | 0.730 | 1.27 (0.68-2.34) | 0.451 | 0.98 (0.70-1.38) | 0.915 |
| Every time | 1 (reference) |  | 1 (reference) |  | 1 (reference) |  |
| **Monkeypox related cognition** | | | | | | |
| **Knowledges of monkeypox** | | | | | | |
| Low (scores 0-5) | 1 (reference) |  | 1 (reference) |  | 1 (reference) |  |
| Moderate (scores 6-11) | 3.24 (2.40-4.37) | <0.001* | 2.20 (1.22-3.96) | 0.009* | 3.71 (2.62-5.26) | <0.001* |
| High (scores12-16) | 6.43 (4.24-9.73) | <0.001* | 6.24 (2.57-15.14) | <0.001* | 6.53 (4.08-10.45) | <0.001* |
| **Knowledges of preventing monkeypox** | | | | | | |
| Low (scores 0-2) | 1 (reference) |  | 1 (reference) |  | 1 (reference) |  |
| Moderate (scores 3-5) | 5.31 (3.95-7.15) | <0.001* | 5.67 (3.10-10.38) | <0.001* | 5.22 (3.71-7.34) | <0.001* |
| High (scores 6-7) | 11.14 (6.76-18.36) | <0.001* | 49.94 (6.80-366.85) | <0.001* | 8.88 (5.25-15.00) | <0.001* |
| **Contact with people and animals in epidemic area** | | | | | | |
| No | 1 (reference) |  | 1 (reference) |  | 1 (reference) |  |
| Yes | 0.47 (0.29-0.74) | 0.001* | 0.40 (0.14-1.08) |  | 0.50 (0.30-0.84) | 0.008* |
| **Similar symptoms in last 2 months**^§^ | | | | | | |
| No | 1 (reference) |  | 1 (reference) |  | 1 (reference) |  |
| Yes | 0.75 (0.47-1.21) | 0.244 | 0.96 (0.37-2.49) | 0.929 | 0.67 (0.39-1.17) | 0.160 |
| **Perceived susceptibility**^¶^ |  |  |  |  |  |  |
| No or not sure | 1 (reference) |  | 1 (reference) |  | 1 (reference) |  |
| Yes | 5.84 (4.46-7.65) | <0.001* | 6.03 (3.49-10.43) | <0.001* | 5.73 (4.20-7.81) | <0.001* |

^*^ *P*<0.05

^†^ This group includes people who have tested negative for HIV and those who have not been tested.

^§^ Six main symptoms of monkeypox were investigated in this questionnaire, including chills and fever, enlarged lymph nodes, single or multiple rashes on the genitals or perianal area or other parts of the body, weak, muscle pain and [headache](javascript:;). Having any of these symptoms in the last two months was defined as “Yes”.

^¶^ All respondents were asked to answer the question “Are you worried about infecting monkeypox?” Answers include “Yes”, “No”, and “Not sure.”

| **Table S3** Multivariable logistic regression of the factors associated with the vaccine acceptance among the Chinese homosexual and bisexual adults | | | | | | |
| --- | --- | --- | --- | --- | --- | --- |
| **Characteristics** | **Total (n=2,618)** | | **People with HIV (n=722)** | | **People without HIV**^†^ **(n=1,896)** | |
|  | aOR (95% CI) | *P* value | aOR (95% CI) | *P* value | aOR (95% CI) | *P* value |
| **Age group (years)** |  |  |  |  |  |  |
| <25 |  |  | 1 (reference) |  |  |  |
| 25-29 |  |  | 0.60 (0.23-1.56) | 0.297 |  |  |
| 30-34 |  |  | 1.84 (0.67-5.04) | 0.234 |  |  |
| 35-39 |  |  | 1.01 (0.35-2.89) | 0.992 |  |  |
| ≥40 |  |  | 1.92 (0.63-5.91) | 0.253 |  |  |
| **Education** |  |  |  |  |  |  |
| High school and below |  |  | 1 (reference) |  |  |  |
| Junior college degree |  |  | 0.68 (0.28-1.66) | 0.399 |  |  |
| Bachelor degree |  |  | 0.40 (0.18-0.89) | 0.024* |  |  |
| Master degree or above |  |  | 0.44 (0.12-1.63) | 0.220 |  |  |
| **Syphilis infection** |  |  |  |  |  |  |
| Yes |  |  | 0.65 (0.35-1.21) | 0.175 |  |  |
| No or not sure |  |  | 1 (reference) |  |  |  |
| **Sexual orientation** |  |  |  |  |  |  |
| Homosexual |  |  |  |  | 1.34 (0.91-1.97) | 0.133 |
| Bisexual |  |  |  |  | 1 (reference) |  |
| **Condom use** |  |  |  |  |  |  |
| Never |  |  | 0.64 (0.18-2.24) | 0.483 |  |  |
| Sometimes |  |  | 2.18 (1.06-4.47) | 0.034* |  |  |
| Every time | 1 (reference) |  | 1 (reference) |  |  |  |
| **Knowledges of monkeypox** |  |  |  |  |  |  |
| Low | 1 (reference) |  |  |  | 1 (reference) |  |
| Moderate | 1.47 (1.04-2.08) | 0.030* |  |  | 1.82 (1.21-2.72) | <0.001* |
| High | 2.03 (1.23-3.34) | 0.005* |  |  | 2.42 (1.36-4.29) | <0.001* |
| **Knowledges of preventing monkeypox** | | | | | | |
| Low | 1 (reference) |  | 1 (reference) |  | 1 (reference) |  |
| Moderate | 3.52 (2.51-4.94) | <0.001* | 7.18 (3.62-14.23) | <0.001* | 3.05 (2.06-4.52) | <0.001* |
| High | 5.32 (2.98-9.47) | <0.001* | 58.34 (7.65-445.01) | <0.001* | 3.60 (1.92-6.75) | <0.001* |
| **Contact with people and animals in epidemic area** | | | | | | |
| Yes | 0.42 (0.25-0.70) | 0.001* | 0.36 (0.11-1.24) | 0.106 | 0.43 (0.24-0.76) | 0.004* |
| No | 1 (reference) |  | 1 (reference) |  | 1 (reference) |  |
| **Perceived susceptibility**^¶^ |  |  |  |  |  |  |
| Yes | 4.37 (3.29-5.80) | <0.001* | 5.37 (2.90-9.94) | <0.001* | 4.17 (3.00-5.79) | <0.001* |
| No or not sure | 1 (reference) |  | 1 (reference) |  | 1 (reference) |  |

^*^ *P*<0.05

^†^ This group includes people who have tested positive for HIV and those who have not been tested.

^§^ Six main symptoms of monkeypox were investigated in this questionnaire, including chills and fever, enlarged lymph nodes, single or multiple rashes on the genitals or perianal area or other parts of the body, weak, muscle pain and [headache](javascript:;). Having any of these symptoms in the last two months was defined as “Yes”.

^¶^ All respondents were asked to answer the question “Are you worried about infecting monkeypox?” Answers include “Yes”, “No”, and “Not sure.”


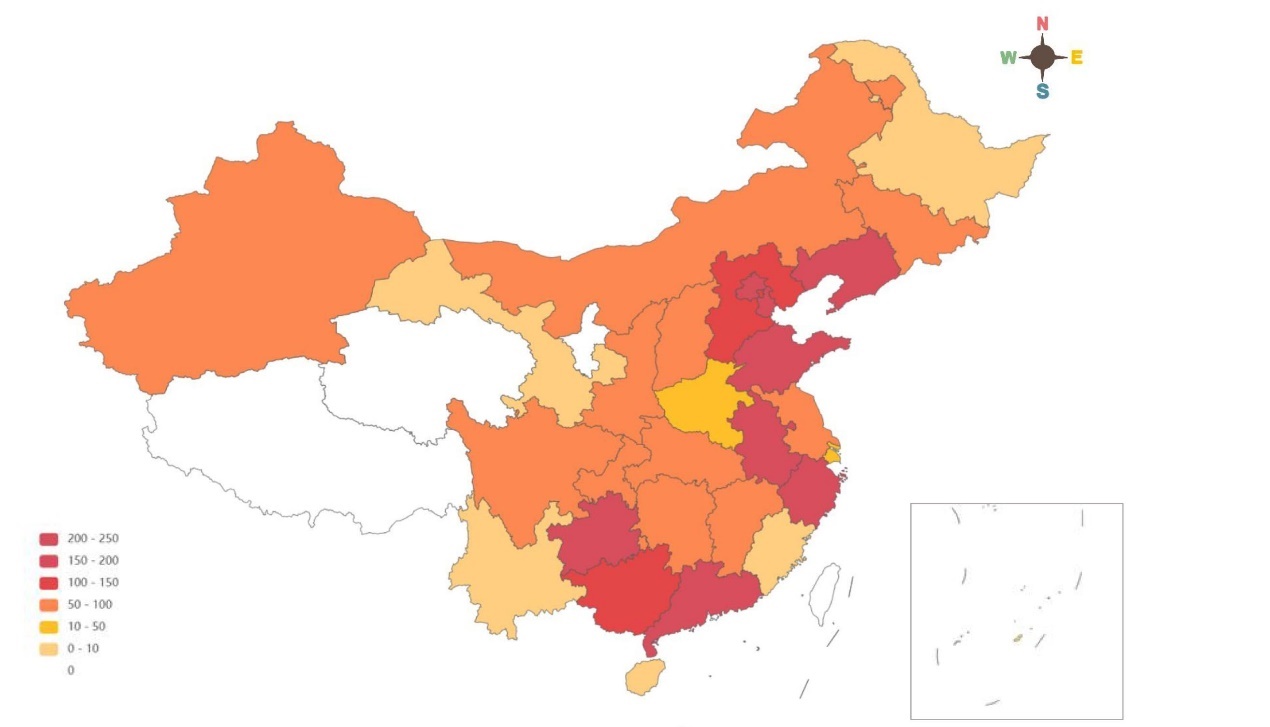
**Figure S1** Provincial distribution map of the included MSM individuals (n=2,618)
